# A ternary complex comprising transportin1, Rab8 and the ciliary targeting signal directs proteins to ciliary membranes

**DOI:** 10.1242/jcs.194019

**Published:** 2016-10-15

**Authors:** Viswanadh Madugula, Lei Lu

**Affiliations:** School of Biological Sciences, Nanyang Technological University, 60 Nanyang Drive, 637551Singapore

**Keywords:** Cilia, Transportin1, Rab8, Ciliary targeting signal

## Abstract

The sensory functions of cilia are dependent on the enrichment of cilium-resident proteins. Although it is known that ciliary targeting signals (CTSs) specifically target ciliary proteins to cilia, it is still unclear how CTSs facilitate the entry and retention of cilium-resident proteins at the molecular level. We found that non-ciliary membrane reporters can passively diffuse into cilia through the lateral transport pathway, and the translocation of membrane reporters through the ciliary diffusion barrier is facilitated by importin binding motifs and domains. Screening known CTSs of ciliary membrane residents uncovered that fibrocystin, photoreceptor retinol dehydrogenase, rhodopsin and retinitis pigmentosa 2 interact with transportin1 (TNPO1) through previously identified CTSs. We further discovered that a new ternary complex, comprising TNPO1, Rab8 and a CTS, can assemble or disassemble under the guanine nucleotide exchange activity of Rab8. Our study suggests a new mechanism in which the TNPO1–Rab8–CTS complex mediates selective entry into and retention of cargos within cilia.

## Abstract (precis)

**Summary:** Transportin1 and Rab8 can simultaneously bind to the ciliary targeting signal in a guanine-nucleotide-dependent manner. The resulting ternary complex can target a membrane protein to cilia.

## INTRODUCTION

Primary cilia (hereafter cilia) are hair-like organelles on the cell surface that can sense diverse environmental cues and initiate corresponding intracellular signaling. Therefore, cilia play important roles in tissue development and homeostasis, and defects in cilia can cause a broad range of human genetic diseases, which are collectively called ciliopathies (Hildebrandt et al*.*, 2011; Basten and Giles, 2013; Falk et al*.*, 2015; Lee et al*.*, 2015). The sensory functions of cilia rely on the presence of a battery of membrane proteins and receptors on the ciliary membrane. How cilium-resident membrane proteins are specifically targeted to cilia is a fundamental question that remains open. Although soluble cargos access the ciliary interior from the cytosol through a soluble diffusion barrier at the opening near the cilium base, membrane cargos can use the following two pathways (Nachury et al., 2010). In the polarized exocytosis pathway, a membrane protein is first packed into a vesicle derived from either the secretory or endocytic pathway. Then, the vesicle specifically fuses to the plasma membrane near the cilium base (periciliary membrane), and the membrane cargo subsequently enters cilia (Papermaster et al., 1985). In contrast, in the lateral transport pathway, cargos at the plasma membrane can directly slide through the ciliary opening to the ciliary membrane without membrane fission or fusion (Hunnicutt et al*.*, 1990; Milenkovic et al*.*, 2009; Leaf and Von Zastrow, 2015). Despite the difference in the site of membrane insertion – the plasma membrane and periciliary membrane for the lateral transport and polarized exocytosis pathways, respectively – cargos of both pathways must cross a membrane diffusion barrier at or near the transition zone before entering the ciliary membrane (Nachury et al*.*, 2010; Verhey and Yang, 2016). The physical and functional existence of the membrane diffusion barrier has been substantiated by morphological data from electron microscopy analyses (Gilula and Satir, 1972) and kinetic data from the fluorescence recovery after photobleaching (FRAP) analyses (Hu et al*.*, 2010; Chih et al*.*, 2012; Leaf and Von Zastrow, 2015). At the molecular level, the B9 complex (also known as the Meckel syndrome or nephronophthisis complex) (Williams et al*.*, 2011; Chih et al*.*, 2011; Lambacher et al*.*, 2016), Septin2 (Hu et al., 2010) and densely packed membrane lipids (Vieira et al., 2006) have been proposed to contribute to the membrane diffusion barrier. However, it is still unclear how membrane cargos selectively cross the barrier and are retained within cilia. Although soluble and membrane diffusion barrier functions can be implemented by the same cellular structures, recent evidence demonstrates that the soluble diffusion barrier is probably imposed by alternative machinery, such as by the cilium-base-localized nucleoporin complex (Kee et al*.*, 2012; Takao et al*.*, 2014).

The targeting of proteins to distinct subcellular compartments is mediated by signals which usually comprise linear and short stretches of amino acids. More than a dozen ciliary targeting signals (CTSs) have been discovered, although the molecular mechanism underlying their targeting is still unknown (Nachury et al*.*, 2010; Hsiao et al*.*, 2012; Madhivanan and Aguilar, 2014). Available data demonstrate that CTSs neither converge to sequence consensus nor share trafficking machinery (Nachury et al*.*, 2010; Madhivanan and Aguilar, 2014). Recent discoveries have revealed an unexpected role of nucleocytoplasmic transport machinery, especially importin-β1 and transportin1 (also known as importin-β2 and TNPO1), in the ciliary targeting of membrane cargos, such as Crumbs3 and retinitis pigmentosa 2 (RP2), and soluble cargos such as KIF17 (Fan et al*.*, 2007, 2011; Dishinger et al*.*, 2010; Hurd et al*.*, 2011; Kee et al*.*, 2012). Both importin-β1 and TNPO1 belong to the β-karyopherin family and are evolutionarily conserved cargo receptors for nucleocytoplasmic trafficking (Marfori et al*.*, 2011; Twyffels et al*.*, 2014; Soniat and Chook, 2015). In nucleocytoplasmic trafficking, the weak and transient interactions between importins and FG-repeats of nucleoporins facilitate crossing of the importin–cargo complex across the diffusion barrier, which is formed by FG-repeats within the nuclear pore complex (Stewart, 2007; Marfori et al*.*, 2011). In this study, we attempted to elucidate the molecular and cellular mechanisms underlying CTS function, as well as their cognate transport machinery for ciliary membrane proteins. We discovered that a new ternary complex, comprising TNPO1, Rab8 and a CTS, can assemble and subsequently disassemble in order to transport ciliary membrane cargos under the regulation of Rab8 guanine nucleotide exchange factors (GEFs).

## RESULTS

### Quantification of ciliary localization by using the cilium:plasma-membrane intensity ratio

**Fig. 1.**
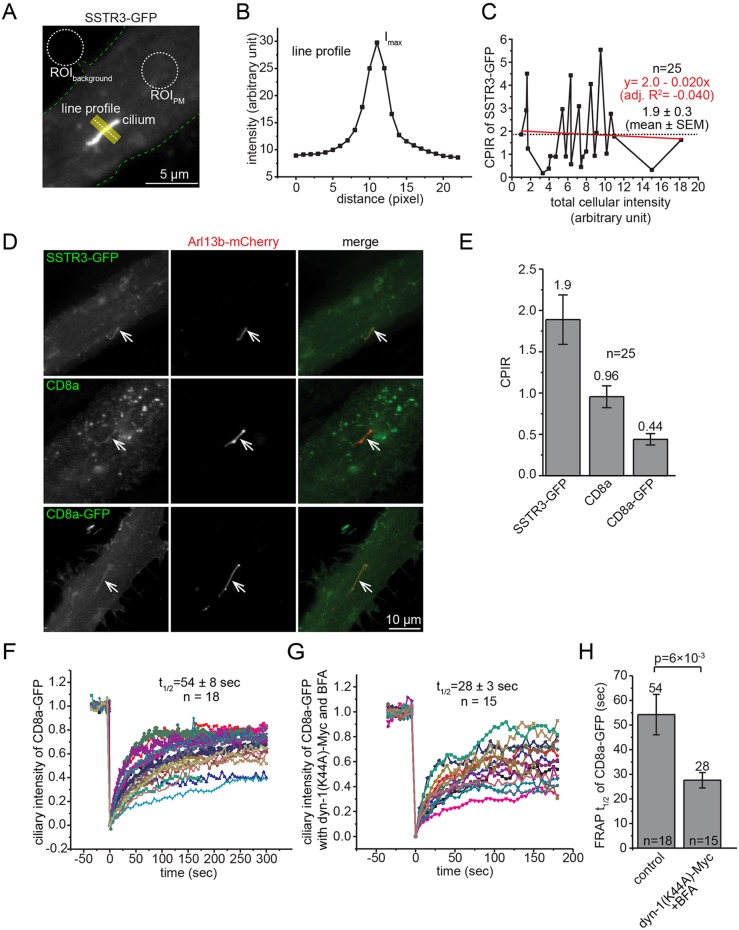
**CD8a can access cilia through the lateral transport pathway.** (A) A schematic diagram illustrating the acquisition of the CPIR of SSTR3–GFP. A ciliated RPE1 cell expressing SSTR3–GFP was imaged. ROIs of the background (ROI_background_) and plasma membrane (ROI_PM_) are shown by dotted circles and were used to calculate I_background_ and I_PM_, respectively. The contour of the cell is marked by dotted green lines. A yellow line (with the width of ∼1 µm) is drawn across the cilium, and the corresponding line intensity profile is shown in B. (B) Intensity profile of the line. I_max_ is the peak intensity. (C) The trend of the CPIR is independent of the expression level. For each RPE1 cell expressing SSTR3–GFP, the CPIR was plotted against the total intensity of the cell. Data points are connected by lines from low to high total intensities of the cell. Dotted horizontal line indicates mean. The trend of data is represented by a linear regression fitting line, which, together with its formula and adjusted R^2^ value (adj. R^2^), is shown in red. (D) CD8a and CD8a–GFP were detected in cilia. RPE1 cells transiently co-expressing Arl13b–mCherry and SSTR3–GFP, CD8a or CD8a–GFP were induced to generate cilia and imaged. Cilia are indicated by arrows. (E) CPIR values of CD8a and CD8a–GFP. *n*=25. The mean is indicated at the top of each column. (F–H) Simultaneous inhibition of secretory and endocytic pathways did not reduce the recovery kinetics during whole cilium FRAP analysis. Ciliated RPE1 cells expressing CD8a–GFP alone (control) (F) or co-expressing CD8a–GFP and dynamin-1(K44A)–Myc [dyn-1(K44A)–Myc] and treated with BFA (G) were subjected to whole cilium FRAP analysis, and FRAP traces are shown. (H) FRAP half lives (t_1/2_). The mean values and number of cells (*n*) are indicated. Error bars are s.e.m. The *P* value was calculated by using the *t*-test.

Ciliary localization is conventionally quantified as the percentage of cilium-positive cells calculated by using fluorescence imaging. However, this method unavoidably introduces bias as the threshold for positive localization is subjectively determined, and the cargo concentration within positive cilia can vary dramatically. Although it is intuitive to adopt the total intensity signal within the cilium as a measure of ciliary localization, such quantification is influenced by not only the cilium length but also the cellular expression level of a ciliary protein, both of which can fluctuate substantially within a population of cells. We established a fluorescence-image-based and ensemble-averaged metric, the cilium to plasma-membrane intensity ratio (CPIR), to quantify the ciliary localization of a membrane protein in cultured mammalian cells. To that end, a line with a width of ∼1 µm was drawn orthogonally across the cilium, and the maximum of the line intensity profile (I_max_) was subsequently obtained (Fig. 1A,B). Surface labeling can be applied to reduce the interference with the quantification of the intensity of the plasma membrane (I_PM_) due to intracellular signals. After acquiring the mean I_PM_ and the background value (I_background_), the CPIR of the membrane protein is defined as (I_max_−I_PM_)/(I_PM_−I_background_). The CPIR indicates the relative enrichment of a membrane protein for the unit length of the cilium by normalizing its expression level at the plasma membrane. Importantly, the trend of CPIR was observed to remain independent of the expression level for >10-fold range (Fig. 1C; Fig. S1A–J). In this study, the CPIR mean from a population of cells has been used to quantitatively indicate the ciliary localization or targeting of a ciliary membrane reporter.

### Plasma-membrane-localized membrane proteins are able to diffuse passively into the ciliary membrane

CD8a, a plasma-membrane-localized type-I transmembrane protein, is conventionally assumed to be non-ciliary and has been previously used as a reporter to study ciliary targeting of membrane proteins (Follit et al., 2010; Jin et al., 2010). Surprisingly, our imaging data always showed a significant ciliary localization of CD8a and CD8a–GFP in RPE1, BSC-1 and IMCD3 cells (Fig. 1D–E; Fig. S2A). We found that the CPIR of CD8a sharply decreased as its cytosolic molecular mass increased through tagging with 0–3 copies of GFP (Fig. S2B–D). CD8a–GFP×2 and CD8a–GFP×3 were essentially undetectable at cilia (Fig. S2D), implying that the ciliary membrane diffusion barrier has a cytosolic size-exclusion limit of 50–100 kDa (considering that CD8a forms a homodimer; Rybakin et al., 2011), similar to the value observed for soluble proteins (Kee et al., 2012).

We initially thought that there could be an uncharacterized CTS in the cytosolic domain of CD8a. However, when both cytosolic and transmembrane domains of CD8a were swapped with the corresponding domains of CD4, a type-I transmembrane protein that is natively expressed in only T cells, a significant ciliary localization of the CD8a–CD4 chimera was also observed (Fig. S2E,G). Furthermore, ciliary localization was also observed for diverse plasma membrane proteins that are not expected to be ciliary residents, such as interleukin 2 receptor α subunit (IL2Rα, a type-I transmembrane protein), GFP–CAAX (lipid-anchored), Vamp5–GFP (tail-anchored), CD59 (glycosylphosphatidylinositol- or GPI-anchored) and endocytosis-defective mutants of Vamp2 and Vamp8 (Miller et al., 2011) (Fig. S2E,G). Supporting our observation, ciliary localization of GFP–GPI and GFP–CEACAM1 has been previously reported in IMCD3 cells (Francis et al., 2011). However, we found that not all plasma membrane proteins localized to cilia. When the cytosolic tail of CD8a was replaced by that of furin or sortilin, surface labeling revealed that these chimeras localized at clathrin-coated pits instead of cilia (Fig. S2F–H). It has been reported that actin binding can retain a membrane protein on the plasma membrane and prevent it from entering cilia (Francis et al., 2011). Collectively, our data demonstrate that the ciliary membrane diffusion barrier is leaky and that plasma membrane proteins, if not restrained by clathrin-coated pits or actin cytoskeleton, can enter ciliary membranes non-selectively.

We subsequently asked how a plasma membrane protein such as CD8a can enter the ciliary membrane. Two pathways are known for the ciliary targeting of membrane cargos – the lateral and polarized exocytosis transport pathways. Since CD8a lacks appropriate sorting signals, it does not undergo polarized secretion or receptor-mediated endocytosis, leaving the lateral transport pathway as the most plausible mechanism. Using whole-cilium FRAP, we measured the half life of CD8a–GFP as 54±8 s (mean±s.e.m, and throughout) (*n*=18) (Fig. 1F,H). To rule out the possible contribution of the polarized exocytosis transport pathway, we inhibited endocytosis by overexpressing dynamin-1(K44A) (van der Bliek et al., 1993) – a GTPase-defective dominant-negative mutant – and secretion through treatment with Brefeldin A (BFA) (Lippincott-Schwartz et al., 2000) (Fig. S1K,L). Under such conditions, we found that the half life of CD8a–GFP became 28±3 s (*n*=15) (Fig. 1G,H). Although we do not have a satisfactory explanation for the increased dynamics at the moment, the observation suggests that CD8a and probably other plasma membrane proteins could adopt the lateral transport pathway to access the ciliary membrane.

### Importin-binding motifs and domains increase ciliary localization of membrane reporters

**Fig. 2.**
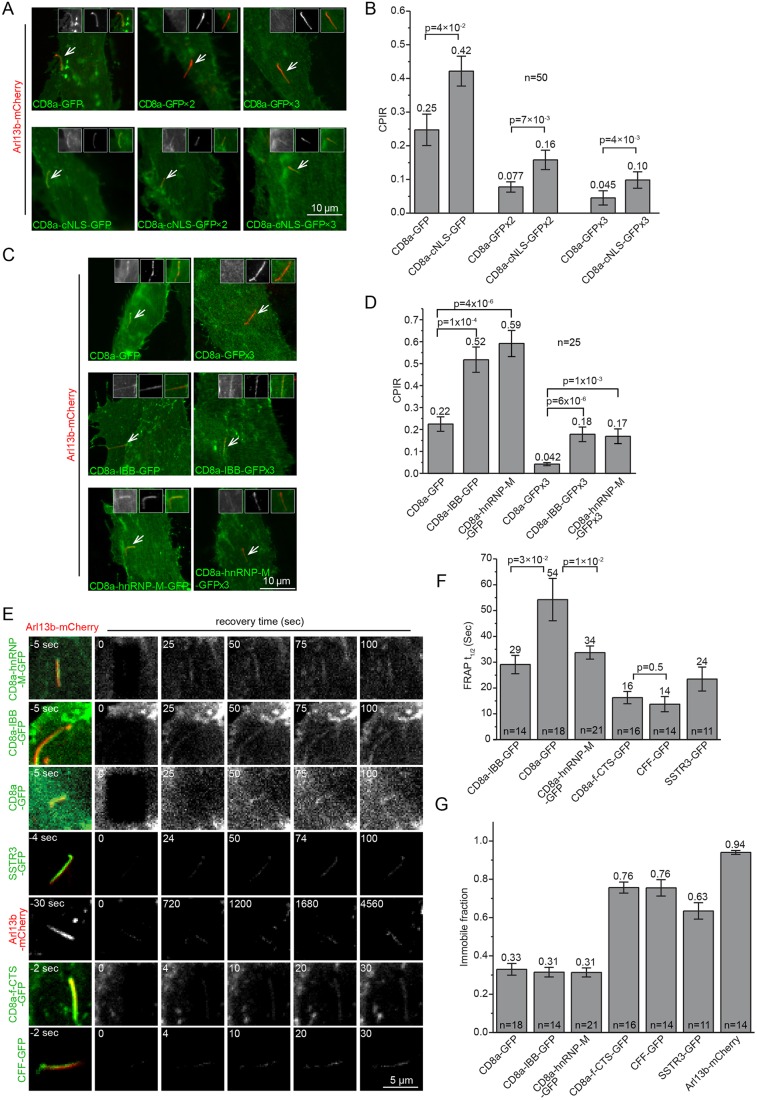
**Importin-binding motifs and domains increase the ciliary localization of membrane reporters.** (A,B) SV40-cNLS increased the ciliary localization of CD8a chimeras tagged with 1–3 GFP molecules. Images showing representative ciliated RPE1 cells co-expressing Arl13b–mCherry and CD8a chimeras. The cilium of interest is indicated by an arrow. The three inserts in each image show the cilium in GFP (monochromatic, left), mCherry (monochromatic, middle) and merge (color, right) channel. The bar graph shows CPIR values of CD8a chimeras. (C,D) IBB and the PY-NLS signal of hnRNP-M increased the ciliary localization of CD8a–GFP or CD8a–GFP×3. The organization of images and bar graph is similar to those described for A,B. (E) Representative two-dimensional time-lapse images of cilia expressing various fluorescence chimeras during whole cilium FRAP analysis. Live ciliated RPE1 cells expressing the indicated fluorescence chimeras were imaged by using a spinning disk confocal microscope. The whole cilium was photobleached at 0 s. Time is indicated at the upper left of each image. The half lives and immobile fractions of FRAP are plotted in F and G, respectively. Both CD8a-f-CTS–GFP and CFF–GFP contain the CTS of fibrocystin. Note that the half life value of CD8a–GFP in Fig. 1H (control) is duplicated here for comparison. The number of cells, *n*, is labeled in each bar graph. Error bars are s.e.m. The mean value is indicated at the top of each column. *P*-values (*t*-test) of selected pairs are denoted.

Because recent studies have revealed the role of importins in ciliary targeting (Fan et al., 2007; Hurd et al., 2011), we quantitatively evaluated various importin binding motifs and domains in targeting CD8a to cilia. The cytosolic tail of CD8a was replaced with the following importin binding motifs and domains: (1) the classic nuclear localization signal (cNLS) of SV40 large T antigen, which binds to the importin-α and importin-β1 heterodimer (Marfori et al., 2011), (2) the importin-β1 binding domain of importin-α (IBB) (Görlich et al., 1996) and (3) the basic PY-NLS (bPY-NLS) motif of hnRNP-M, which binds to TNPO1 (Lee et al., 2006). The three motifs and domains significantly increased the CPIRs of CD8a–GFP derivative reporters (Fig. 2A–D) (*P*<0.05 by *t*-test), demonstrating that importin binding motifs and domains can increase the ciliary localization of membrane reporters.

We subsequently employed whole cilium FRAP analysis to study the role of importin binding motifs and domains in ciliary localization of CD8a (Fig. 2E–G). When fused to bPY-NLS or IBB, the CD8a reporter displayed a significantly reduced half life of 34±3 s (*n*=21) or 29±4 s (*n*=14), respectively, lower than that of CD8a–GFP [54±8 s (*n*=18); *P*=0.01 compared to bPY-NLS and *P*=0.03 compared to IBB, respectively] (Fig. 2F; Fig. S1M,N). Their significantly lower half lives imply the facilitated crossing of the membrane diffusion barrier, consistent with the role of importins in nucleocytoplasmic trafficking (Stewart, 2007; Marfori et al*.*, 2011). Notably, fibrocystin and SSTR3, two ciliary membrane residents, also have shorter half lives than CD8a–GFP (Fig. 2F; Fig. S1O–Q). However, the immobile fraction values of both IBB (0.31) and bPY-NLS (0.31) chimeras were similar to that of CD8a-GFP (0.33) (Fig. 2G). In contrast, ciliary membrane residents, such as SSTR3, Arl13b and fibrocystin, displayed much higher immobile fraction values (0.63–0.94) (Fig. 2G), as previously reported (Hu et al., 2010; Larkins et al., 2011). Collectively, our data suggest that importins promote the ciliary localization of membrane proteins by facilitating entry into instead of retention within cilia.

### CTSs of fibrocystin, prRDH and rhodopsin can interact with TNPO1

**Fig. 3.**
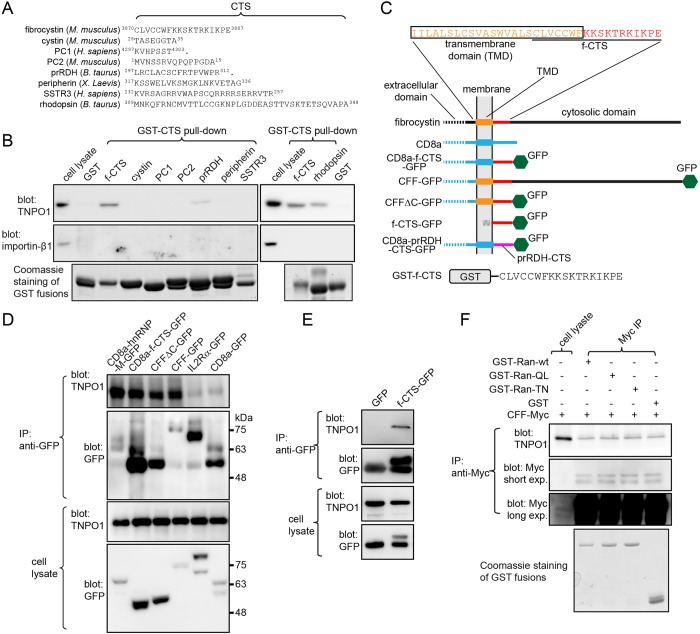
**f-CTS interacts with TNPO1.** All cell lysates were from HEK293T cells. (A) Sequences of CTSs used in screening. Positions of the first and last amino acids are indicated by numbers. ‘.’ indicates the end of the coding sequence. (B) Screening revealed CTSs of fibrocystin, prRDH and rhodopsin interacted with TNPO1 but not with importin-β1. Cell lysates were incubated with various bead-immobilized GST-fused CTSs and the pull down was blotted for TNPO1 and importin-β1. (C) A schematic diagram illustrating various fusion chimeras of fibrocystin and prRDH used in this study. TMD, transmembrane domain. The amino acid sequences of the TMD and f-CTS are indicated and represented by rectangles and lines, respectively, of the same color in the diagram. Black and blue annotations denote corresponding amino acid sequences from fibrocystin and CD8a, respectively. Note that f-CTS–GFP associates with the membrane through the palmitoyl group. (D) CD8a chimeras of fibrocystin specifically co-immunoprecipitate endogenous TNPO1. Cell lysates expressing various GFP-tagged chimeras were subjected to immunoprecipitation (IP) using an anti-GFP antibody, and co-immunoprecipitated material was blotted for TNPO1 and GFP. CD8a-hnRNP-M–GFP is a positive control. IL2Rα–GFP and CD8a–GFP are negative controls and showed background binding. CD8a chimeras display complex band patterns owing to O-glycosylation. In selected gel blots, numbers at the right indicate molecular weight markers in kDa. (E) f-CTS–GFP specifically co-immunoprecipitated endogenous TNPO1. The co-immunoprecipitation experiment was performed similar to that described for D. GFP is a negative control. (F) The interaction between fibrocystin (CFF) and TNPO1 is probably not regulated by Ran GTPase. Cell lysates expressing CFF–Myc were subjected to co-immunoprecipitation using an anti-Myc antibody in the presence of the following recombinant proteins: GST (negative control), GST–Ran-wt, GST–Ran-QL or GST–Ran-TN. The co-immunoprecipitated TNPO1 was subsequently blotted. Two images from short and long exposure (exp.) of the same anti-Myc blot shows immunoprecipitated and cell lysate CFF–Myc, respectively.

To test the hypothesis that ciliary membrane residents can utilize importin-β1 or TNPO1 for ciliary targeting, we screened eight CTSs that are known to be sufficient for ciliary targeting of membrane reporters. These CTSs, ranging from 7 to 40 residues, were from fibrocystin (Follit et al., 2010), cystin (Tao et al., 2009), polycystin-1 (PC1) (Ward et al., 2011), polycystin-2 (PC2) (Geng et al., 2006), prRDH (also known as RDH8) (Luo et al., 2004), peripherin (Tam et al., 2004), SSTR3 (Jin et al., 2010) and rhodopsin (Tam et al., 2000) (Fig. 3A). GST-fused CTSs of fibrocystin (hereafter f-CTS), prRDH and rhodopsin specifically pulled down endogenous TNPO1 but not importin-β1 (Fig. 3B). Examination of primary sequences of the three CTSs did not reveal a PY-NLS consensus motif, which is known to be recognized by TNPO1 (Lee et al., 2006). We first focused on f-CTS for detailed characterization owing to its consistently strong interaction with TNPO1.

### f-CTS specifically interacts with TNPO1

Fibrocystin is a type-I transmembrane protein of more than 400 kDa, the majority of which forms an extracellular domain. Lacking the full-length fibrocystin construct, we generated a fibrocystin mimetic fusion protein – named CD8a luminal domain, fibrocystin transmembrane domain and fibrocystin cytosolic domain (CFF) – by replacing its long luminal domain with the corresponding domain of CD8a (Fig. 3C). CFFΔC was generated by deleting the C-terminus of CFF so that it contained only f-CTS in its cytosolic domain, whereas CD8a-f-CTS was made by replacing the cytosolic domain of CD8a with f-CTS (Fig. 3C). The three chimeras displayed high CPIR values (∼8) in ciliated RPE1 cells (Fig. S3A–B). f-CTS–GFP is palmitoylated in cytosol and behaves as a ciliary membrane protein (Follit et al., 2010). When expressed in HEK293T cells, GFP-tagged CFF, CFFΔC, CD8a-f-CTS and f-CTS specifically co-immunoprecipitated endogenous TNPO1 (Fig. 3D,E). The four conserved residues within f-CTS, KTRK, which have been reported to be essential for ciliary localization and Rab8 interaction of f-CTS (Follit et al., 2010), were also found to be essential for the f-CTS–TNPO1 interaction (Fig. S3B,C). Therefore, we conclude that fibrocystin interacts with TNPO1 through f-CTS. Although f-CTS has been previously shown to be sufficient for ciliary targeting (Follit et al., 2010), using CFF and its KTRK mutant, we further demonstrated that it is necessary for the targeting (Fig. S3A). Taken together with our findings that the FRAP half-life, immobile fraction and CPIR of CD8a-f-CTS are similar to those of CFF (Fig. 2F,G; Fig. S3B), it seems that all ciliary targeting properties of fibrocystin might be attributed to the f-CTS.

### The interaction between f-CTS and TNPO1 is probably not regulated by Ran GTPase

Serial truncations further revealed that the region from residue 316 to 539 of TNPO1 is essential for its interaction with f-CTS, whereas the Ran-binding region at the N-terminus is dispensable (Fig. S3D,E). It is known that the importin–cargo complex can be disassembled through Ran-GTP binding to importin (Marfori et al*.*, 2011; Twyffels et al*.*, 2014; Soniat and Chook, 2015). Using recombinant GST–Ran wild type (wt), and T24N (hereafter referred to as TN; GDP-bound mutant) and Q69L (hereafter referred to as QL; GTP-bound mutant) mutants, we found that TNPO1 primarily interacted with GST–Ran-QL (Fig. S3F), consistent with findings previous studies (Izaurralde et al*.*, 1997; Siomi et al*.*, 1997; Hurd et al*.*, 2011). However, a saturating amount of GST–Ran-QL did not reduce the interaction between CFF and TNPO1 (Fig. 3F). Furthermore, the CPIR of f-CTS–GFP was unaffected by overexpressing Ran-QL (Fig. S3G–I). Therefore, Ran-GTP probably does not regulate TNPO1-dependent trafficking of fibrocystin, in contrast to its role in the interaction between TNPO1 and RP2 (Hurd et al., 2011). Our data are consistent with previous findings that TNPO1-mediated cargo binding and trafficking can be independent of Ran GTPase (Ribbeck et al*.*, 1999; Lowe et al*.*, 2015).

### TNPO1 is essential for the ciliary targeting of fibrocystin, rhodopsin and prRDH

**Fig. 4.**
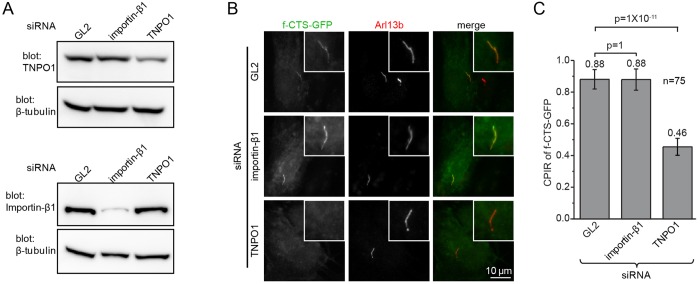
**Knockdown of TNPO1 but not of importin-β1 reduces the ciliary targeting of fibrocystin.** (A) TNPO1 and importin-β1 are specifically knocked down by the relevant siRNAs in RPE1 cells. GL2 siRNA is a negative control. (B,C) RPE1 cells were subjected to knockdown by siRNAs targeting GL2, importin-β1 and TNPO1. Cells were subsequently transfected to express f-CTS–GFP. Images and CPIR values were acquired after induction of ciliogenesis. In each image, the cilium of interest is enlarged and boxed at the upper right corner. Data in C were from *n*=75 cells. Error bars are s.e.m. The mean value is indicated at the top of each column. *P*-values (*t*-test) of selected pairs are denoted.

We quantified the ciliary localization of f-CTS when endogenous importin-β1 or TNPO1 was knocked down. We observed that the depletion of TNPO1 reduced ciliogenesis (Fig. S3J–L). In the remaining ciliated cells, the CPIR of f-CTS–GFP also significantly decreased in comparison to that of the control (Fig. 4A–C; Fig. S3M,N). In contrast, the CPIR remained the same as that of the control upon the depletion of importin-β1 (Fig. 4A–C). Similarly, depletion of TNPO1 resulted in significantly reduced CPIR values for GFP-tagged full-length rhodopsin and CD8a-prRDH-CTS (Fig. S3O–Q). Therefore, in addition to previously reported findings regarding RP2 (Hurd et al., 2011), fibrocystin, rhodopsin and prRDH also require TNPO1 for ciliary targeting.

### TNPO1, Rab8 and f-CTS form a ternary complex that is regulated by the guanine nucleotide binding status of Rab8

**Fig. 5.**
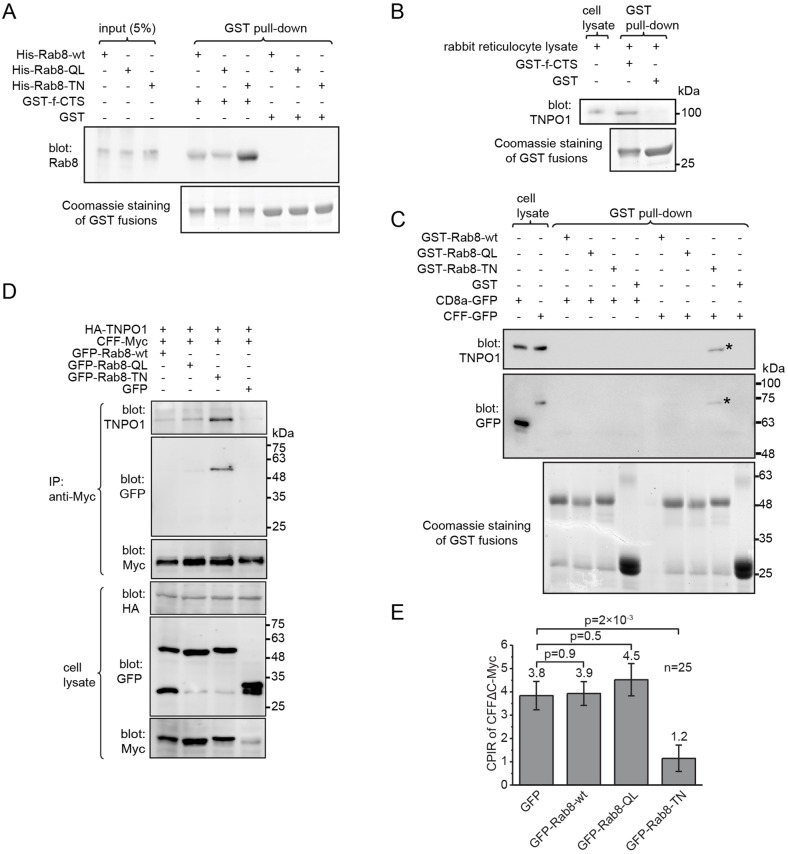
**TNPO1, Rab8 and f-CTS form a ternary complex.** All cell lysates were from HEK293T cells. (A) f-CTS directly interacted with the GDP-locked Rab8 mutant (Rab8-TN). Bead-immobilized GST–f-CTS or GST was incubated with purified His–Rab8-wt, His–Rab8-QL and His–Rab8-TN. The protein pulled down was blotted with an anti-Rab8 antibody. (B) The interaction between f-CTS and TNPO1 should be direct. Bead-immobilized GST–f-CTS specifically pulled down TNPO1 from rabbit reticulocyte lysate, which contains endogenous TNPO1 but not Rab8 (Fig. S4B). (C) Rab8-TN indirectly interacted with TNPO1 through fibrocystin. Bead-immobilized GST–Rab8 fusion proteins were incubated with cell lysate expressing CD8a–GFP or CFF–GFP, and the material pulled down was blotted for TNPO1 and GFP chimeras. * indicates the specific protein band. (D) Rab8-TN promoted the interaction between fibrocystin and TNPO1. The cell lysates co-expressing HA–TNPO1, CFF–Myc and one of the following chimeras, GFP–Rab8-wt, GFP–Rab8-QL, GFP–Rab8-TN and GFP, was subjected to immunoprecipitation using anti-Myc antibody and co-immunoprecipitated TNPO1 and GFP chimeras were blotted. (E) Overexpression of the Rab8 GDP-locked mutant reduced the ciliary localization of fibrocystin. CPIR values of CFFΔC–Myc in ciliated RPE1 cells co-expressing CFFΔC–Myc and one of the following chimeras, GFP–Rab8-wt, GFP–Rab8-QL, GFP–Rab8-TN and GFP. The mean value is indicated at the top of each column. *n*=25. Error bars are s.e.m. *P*-values (*t*-test) of selected pairs are denoted. In all gel blots, numbers at the right indicate the molecular weight markers in kDa.

f-CTS has been reported to preferentially interact with the GDP-bound mutant of Rab8 (Follit et al., 2010). After confirming the interaction (Fig. S4A), we further demonstrated that GST–f-CTS directly interacted with Rab8-GDP using purified His–Rab8-wt, His–Rab8-QL (GTP-bound mutant) and His–Rab8-TN (GDP-bound mutant) (Fig. 5A). We subsequently asked how TNPO1, Rab8 and f-CTS interact with each other. To test whether Rab8 is necessary for the interaction between f-CTS and TNPO1, we took advantage of our observation that rabbit reticulocyte lysate contains endogenous TNPO1 but not Rab8 (Fig. S4B). GST–f-CTS pulled down a substantial amount of TNPO1 from rabbit reticulocyte lysate (Fig. 5B), demonstrating that the interaction between f-CTS and TNPO1 can be direct and independent of Rab8. To test whether Rab8-GDP directly interacts with TNPO1, we used bead-immobilized GST–Rab8 to pull down endogenous TNPO1 in the presence of co-expressed CFF or CD8a, and we found that Rab8-TN interacted with TNPO1 only in the presence of GFP-tagged CFF and not in the presence of CD8a (Fig. 5C). Similarly, Myc–TNPO1 co-immunoprecipitated GFP–Rab8-TN in the presence of HA-tagged CFF but not in the presence of CD8aΔcyto (Fig. S4C). Collectively, our results suggest that f-CTS could simultaneously engage both Rab8–GDP and TNPO1, resulting in the formation of a ternary complex.

To investigate the role of Rab8 in the assembly of this complex, we co-expressed three proteins in cells – HA–TNPO1, CFF–Myc and the GFP–Rab8 mutants or GFP (negative control) (Fig. 5D). We found that CFF–Myc co-immunoprecipitated GFP–Rab8-TN, but not GFP–Rab8-QL or GFP–Rab8-wt (Fig. 5D), consistent with our results shown in Fig. 5A and Fig. S4A. Although TNPO1 was detected in all co-immunoprecipitations using CFF–Myc as the bait, almost threefold more TNPO1 was co-immunoprecipitated in the presence of Rab8-TN than in the presence of Rab8-QL or Rab8-wt (Fig. 5D). It seems that endogenous or overexpressed Rab8-wt did not contribute to the binding between f-CTS and TNPO1 under our experimental conditions and, therefore, retrospectively validated the direct interaction between f-CTS and TNPO1 in our study (Fig. 3; Fig. S3C,E). Although f-CTS can interact with TNPO1 independently of Rab8, our finding that Rab8-GDP instead of Rab8-GTP greatly promoted their interaction implies that the ternary complex can be weakened or disassembled through the guanine nucleotide exchange of Rab8 from GDP to GTP.

We next examined the ciliary localization of CFFΔC under the overexpression of Rab8 mutants. As previously reported (Nachury et al., 2007), we observed that Rab8-TN expression impaired ciliogenesis. In ciliated cells expressing Rab8, the CPIR of CFFΔC decreased significantly in the presence of Rab8-TN compared to that in the presence of GFP, Rab8-wt and Rab8-QL (Fig. 5E; Fig. S4D), confirming the essential role of Rab8 in the ciliary targeting of f-CTS (Follit et al*.*, 2010). Because Rab8-TN is the GDP-locked mutant, GDP to GTP exchange of Rab8 and the ensuing disassembly of the ternary complex is essential for the retention of fibrocystin within cilia. It is possible that, without the guanine-nucleotide-exchange-induced disassembly, the imported TNPO1–Rab8-TN–f-CTS ternary complex can undergo the reverse pathway, export translocation, to the plasma membrane, therefore greatly reducing the CPIR of f-CTS.

### The CTSs of prRDH, rhodopsin and RP2 can form similar ternary complexes with Rab8 and TNPO1

**Fig. 6.**
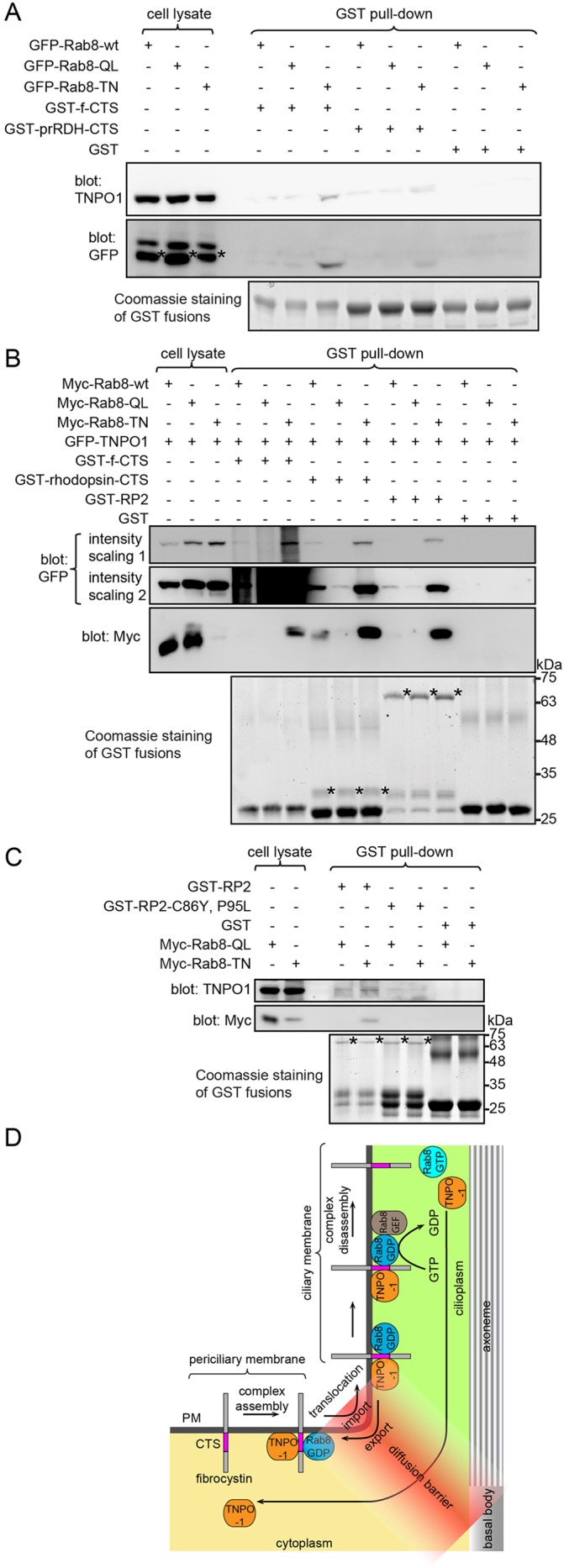
**The CTS of RP2, prRDH or rhodopsin can form similar ternary complexes with Rab8 and TNPO1.** (A) Rab8-TN increased the binding of prRDH-CTS to TNPO1. HEK293T cell lysates expressing GFP–Rab8-wt, GFP–Rab8-QL or GFP–Rab8-TN were subjected to pull down with bead-immobilized GST–prRDH-CTS, GST–f-CTS (positive control) and GST (negative control), and the material pulled down was blotted for GFP–Rab8 and endogenous TNPO1. * denotes the band specific to GFP–Rab8. (B) Rab8-TN increased the binding of rhodopsin-CTS and RP2 to TNPO1. HEK293T cell lysates co-expressing GFP–TNPO1 and Myc–Rab8-wt, Myc–Rab8-QL or Myc–Rab8-TN was subjected to pull down by bead-immobilized and GST-tagged f-CTS (positive control), GST–rhodopsin-CTS, GST–RP2 and GST (negative control), and the material pulled down was blotted for Myc–Rab8 and GFP–TNPO1. * denotes the corresponding GST-fusion protein used as the bait for the pull down. The intensity scaling of the same anti-GFP blot is adjusted to show both bright (intensity scaling 1) and weak bands (intensity scaling 2). (C) The interaction among RP2, Rab8-TN and TNPO1 was abolished by C86Y and P95L mutations of RP2. HEK293T cell lysates expressing Myc–Rab8-wt, Myc–Rab8-QL or Myc–Rab8-TN were subjected to pull down by bead-immobilized GST–RP2, GST–RP2-C86Y, P95L and GST (negative control), and the material pulled down was blotted for Myc–Rab8 and endogenous TNPO1. * denotes the corresponding GST-fusion protein used as the bait for the pull down. In selected gel blots, numbers at the right indicate the molecular weight markers in kDa. (D) Model of ciliary targeting of fibrocystin. See the main text for the description.

We wondered whether other TNPO1-interacting ciliary membrane residents can assemble similar ternary complexes with Rab8 and TNPO1. To that end, we first expanded our study to the CTSs of prRDH and rhodopsin, which were positive hits in our initial screening (Fig. 3B). The involvement of Rab8 in the ciliary targeting of rhodopsin has been previously documented (Moritz et al., 2001; Wang et al., 2012). Indeed, we found that Rab8-TN but not Rab8-QL promoted the pull down of TNPO1 by the GST-fused CTSs of prRDH or rhodopsin (Fig. 6A,B). We next tested the peripheral membrane protein RP2, which is known to interact with TNPO1 for its ciliary targeting (Hurd et al., 2011). Pull down of TNPO1 by GST–RP2 in the presence of Rab8-TN was substantially greater than that in the presence of Rab8-QL (Fig. 6B). When the predominant CTS of RP2 was compromised by C86Y and P95L mutations (Hurd et al., 2011), the interaction among RP2, TNPO1 and Rab8-TN was greatly attenuated (Fig. 6C). Lastly, we found that GFP-tagged RP2, CD8a-prRDH-CTS or full-length rhodopsin was specifically pulled down together with TNPO1 by GST–Rab8-TN (Fig. S4E), therefore suggesting that RP2, prRDH and rhodopsin could assemble similar ternary complexes with Rab8 and TNPO1 through their CTSs.

Our finding prompted us to re-examine our initial screen of CTSs in Fig. 3B because certain CTS–TNPO1 interactions can take place only in the presence of Rab8-TN. However, we found that, except for the CTSs of fibrocystin, prRDH and rhodopsin, the remaining CTSs of our initial screen interacted with neither TNPO1 nor Rab8 in the presence of overexpressed Rab8-TN (Fig. S4F), suggesting that the utilization of Rab8 and TNPO1 as ciliary transport machinery could be specific to certain ciliary membrane proteins.

## DISCUSSION

The plasma membrane and ciliary membrane share the same membrane sheet, yet their proteins and lipids do not freely mix owing to the membrane diffusion barrier at the cilium base (Nachury et al*.*, 2010; Hsiao et al*.*, 2012; Madhivanan and Aguilar, 2014; Verhey and Yang, 2016). It is, however, not understood how ciliary membrane residents cross the membrane diffusion barrier and achieve their retention within cilia. We demonstrated that plasma membrane proteins can passively diffuse across the membrane diffusion barrier to cilia, possibly through the lateral transport pathway. Similar to cilia, the inner nuclear membrane (INM) is in direct continuity with the outer nuclear membrane (ONM) and the endoplasmic reticulum (ER), and the distinct composition of the INM is maintained by nuclear pore complexes, which function as membrane diffusion barriers between the INM and ONM (Hetzer et al., 2005). Our results on cilia parallel what we know about the INM because ER membrane proteins can also passively and laterally diffuse to the INM, but they are retained there to a lesser degree than bona fide INM residents (Zuleger et al., 2012). Two mechanisms have been proposed for targeting to the INM: retention and selective entry mechanisms (Katta et al., 2014). CTSs could adopt two similar mechanisms. (1) The retention mechanism prevents ciliary cargos from exiting cilia. The retention can be mediated by the specific binding of CTSs to their cognate ciliary receptors such as BBSome (Jin et al., 2010) and axonemal microtubule (Fan et al*.*, 2004; Kovacs et al*.*, 2008; Francis et al*.*, 2011). Paradoxically, most known ciliary membrane residents are highly mobile within cilia (Hu et al*.*, 2010; Chih et al*.*, 2011; Breslow et al*.*, 2013; Ye et al*.*, 2013). In contrast to ciliary membrane residents, INM proteins are largely immobile (Ellenberg et al., 1997). (2) In the selective entry mechanism, transport receptors selectively facilitate the crossing of ciliary residents through the diffusion barrier by binding to their CTSs. Studies have shown that importins can act as ciliary transport receptors (Fan et al*.*, 2007; Dishinger et al*.*, 2010; Hurd et al*.*, 2011), similar to their roles in the nucleocytoplasmic trafficking (Stewart, 2007; Marfori et al*.*, 2011; Twyffels et al*.*, 2014; Soniat and Chook, 2015).

Our FRAP data suggest that importins could promote the selective entry of cilia by facilitating the crossing of the membrane diffusion barrier without increasing the ciliary retention of cargos. We identified four native ciliary membrane residents – fibrocystin, prRDH, rhodopsin and RP2 – that specifically form a previously unidentified ternary complex with TNPO1 and Rab8-GDP through their CTSs. It has been reported that Rab8-GTP is enriched whereas Rab8-GDP is depleted in cilia (Nachury et al., 2007). The Rab8-GTP gradient, which is probably maintained by the polarized ciliary localization of Rab8 GEFs – Rabin8 (Hattula et al., 2002) and RPGR (Murga-Zamalloa et al., 2010). Our findings reveal a new molecular and cellular role of Rab8 and TNPO1 in non-vesicular ciliary trafficking, and the following model is conceivable for the ciliary targeting of membrane cargos such as fibrocystin (Fig. 6D). First, fibrocystin can follow either lateral transport or polarized endocytosis pathways to the periciliary membrane. Next, near the basal body, Rab8-GDP can be released from its GDP-dissociation inhibitor (GDI) by Dzip1, the basal-body-localized GDI displacement factor for Rab8 (Zhang et al., 2015). The association between f-CTS and Rab8-GDP further recruits TNPO1 to assemble the ternary import complex. Then, facilitated by TNPO1, the complex translocates across the membrane diffusion barrier (selective entry mechanism). After the import translocation, the GDP moiety of Rab8 is exchanged for GTP through cilium-localized GEFs, and the ternary complex subsequently disassembles therefore releasing free fibrocystin to the ciliary membrane. Lastly, with the export of TNPO1, the exit of fibrocystin to the plasma membrane is prohibited by the membrane diffusion barrier, hence providing a strong retention mechanism to confine the dynamically diffusive movement of fibrocystin within the ciliary membrane.

It is possible that the guanine nucleotide exchange of Rab8 is the rate-limiting step. Consequently, newly imported TNPO1–Rab8–CTS ternary complexes can exit to the plasma membrane through a reversible pathway – export translocation – hence resulting in a rapid but small fraction of recovery during whole-cilium FRAP (Fig. 2F,G). Our model bears similarity to the non-vesicular Ran-GTPase-dependent nucleocytoplasmic transport pathway, in which Ran-GTP binds to importin to disassemble importin–cargo complexes in the nucleus. The Ran-GTP gradient, which is maintained by its nucleus-localized GEF and cytoplasm-localized GTPase-activating protein (GAP), drives nuclear trafficking directionally (Stewart, 2007).

It is tempting to speculate that Rabs and importins cooperatively target cargos to cilia. Supporting this view, Rab23 and TNPO1 have been reported to potentially assemble into a complex that targets KIF17 to cilia (Lim and Tang, 2015). Importins possess repeats of domains that can form diverse interfaces to engage a large repertoire of cargos (Marfori et al*.*, 2011; Twyffels et al*.*, 2014; Soniat and Chook, 2015). Therefore, more ciliary residents are expected to assemble into ternary complexes with Rabs and importins for ciliary targeting. It could be informative to systematically screen ciliary residents for their Rab-dependent interaction with importins.

## MATERIALS AND METHODS

### DNA plasmids

Please see Table S1 for DNA plasmids used in this study. All constructs were confirmed by DNA sequencing.

### Knockdown

The following siRNA oligonucleotides were purchased from Dharmacon: GL2 (5′-CGUACGCGGAAUACUUCGA-3′), siRNA smart pool targeting importin-β1 (#L-017523-00-005) (5′-GAACCAAGCUUGAUCUGUU-3′, 5′-GCUCAAACCCCACUAGUUAUA-3′, 5′-GACGAGAAGUCAAGAACUA-3′, 5′-GGGCGGGAGAUCGAAGACUA-3′) and siRNA smart pool targeting TNPO1 (#L-011308-00-005) (5′-GCAAAGAUGUACUCGUAAG-3′, 5′-GUAUAGAGAUGCAGCCUUA-3′, 5′-GUAAAUACCAGCAUAAGAA-3′ and 5′-GCAAAUGUGUAUCGUGAUG-3′). siRNAs were transfected into RPE1 cells using Lipofectamine 2000 according to the manufacturer's protocol. For the expression of exogenous proteins after knockdown, transfections were conducted 24 h after siRNA transfection. At 48 h after the transfection of siRNA, cells were serum-starved for another 48 h before immunofluorescence labeling was performed.

Endogenous TNPO1 was also depleted by performing lentivirus-mediated transduction of shRNA. 293FT cells were seeded on 0.01% poly-L-lysine-coated 6-well plates. At 60–70% confluence, cells were transfected with packaging plasmids: pLP1, pLP2, pLP/VSVG (Invitrogen) and lentiviral shRNA construct targeting TNPO1 in the ratio of 2:1:1:4 using Lipofectamine 2000. After 18 h of transfection, the medium was replaced with new. After 36–48 h of transfection, the virus-containing medium was collected and filtered to remove cell debris. For shRNA-mediated knockdown of TNPO1, the lentivirus filtrate was immediately incubated with RPE1 cells for 12 h, followed by a second infection with fresh filtrate for another 12 h. Cells were selected in puromycin to enrich lentivirus-infected cells. This stable pool of cells was seeded on coverslips and transfected to express f-CTS–GFP. After the induction of cilia formation through serum starvation, cells were processed for immunofluorescence labeling.

### Antibodies

Antibodies against the following proteins were used and are commercially available (WB and IF stand for western blot and immunofluorescence, respectively): acetylated α-tubulin (Sigma, #6-11B-1, 1:5000 for WB), α-tubulin (Santa Cruz, #sc8035, 1:1000 for WB), β-tubulin (Santa Cruz, #sc5274, 1:1000 for WB), GAPDH (Santa Cruz, #sc25778, 1:1000 for WB), GFP (mouse monoclonal) (Santa Cruz, #sc9996, 1:1000 for WB), GFP (rabbit polyclonal) (Santa Cruz, #sc8334, 1:3000 for WB), importin-β1 (Abcam, #ab2811, 1:3000 for WB), TNPO1 (Abcam, #ab10303, 1:3000 for WB), Myc (Santa Cruz, #sc40, 1:1000 for WB, 1:200 for IF), CD8a (Developmental Studies Hybridoma Bank, clone OKT8, 1:200 for IF), IL2Rα (ATCC, clone 2A3A1H, 1:200 for IF), Rab8 (BD biosciences, #610844, 1:1000 for WB) and horseradish peroxidase (HRP)-conjugated anti-HA antibody (GeneScript, #A-00169, 1:1000 for WB). HRP-conjugated goat anti-mouse and anti-rabbit IgG antibodies were purchased from Bio-Rad. HRP-conjugated protein A was purchased from Abcam. Alexa-Fluor-conjugated goat anti-mouse and anti-rabbit IgG antibodies (1:500 for IF) were purchased from Invitrogen.

### Cell culture and transfection

hTERT RPE1 and IMCD3 cells were maintained in Dulbecco's modified Eagle's medium (DMEM) and Ham's F12 mixture medium supplemented with 10% fetal bovine serum at 37°C under 5% CO_2_. HeLa, BSC-1, HEK293T and 293FT cells were cultured in high-glucose DMEM supplemented with 10% fetal bovine serum at 37°C under 5% CO_2_. HeLa and HEK293T cells were transfected using polyethylenimine (Polysciences, Inc.). RPE1, IMCD3, 293FT and BSC-1 cells were transfected using Lipofectamine 2000 (Invitrogen). Transfection was performed when cells reached 70–80% confluence, according to a standard protocol. To induce ciliogenesis after the overexpression or knockdown of target proteins by transfection, cells were serum starved by incubating in DMEM. Typical starvation times for RPE1, IMCD3 and BSC-1 cells were 2, 2 and 5 days, respectively.

### Purification of GST fusion proteins

GST-fused proteins were purified as previously described (Mahajan et al., 2013; Zhou et al., 2013).

### Purification of His-tagged Rab8 fusion proteins

pET30ax DNA plasmids encoding His-tagged Rab8-wt, Rab-TN and Rab-QL were transformed into BL21 *Escherichia coli* cells. Transformed bacteria were induced, pelleted and lysed as described previously for the purification of GST fusion proteins. The lysate was subjected to centrifugation at 13,000 ***g*** for 30 min, and the supernatant was incubated with pre-washed Ni-NTA agarose beads (QIAGEN) in the presence of 10 mM imidazole at 4°C for 2 h. After beads had been washed with the buffer containing 20 mM HEPES pH 7.3, 200 mM KCl, 10% glycerol and 25 mM imidazole, the bound protein was eluted with the elution buffer containing 20 mM HEPES pH 7.3, 200 mM KCl, 250 mM imidazole, 10% glycerol and 1 mM DTT. The eluted protein was dialyzed and concentrated.

### Generation of a polyclonal antibody against Arl13b

His-tagged Arl13b-C-ter was purified under denaturing conditions using 8 M urea, as previously described (Mahajan et al., 2013). The denatured protein was used to immunize rabbits, and anti-sera were collected by Genemed Synthesis Inc. To purify the Arl13b antibody from the anti-serum, GST–Arl13b-C-ter on glutathione Sepharose beads were prepared as previously described (Mahajan et al., 2013; Zhou et al., 2013) and incubated with dimethyl pimelimidate (Sigma) in 200 mM sodium borate solution pH 9.0 to cross-link the fusion protein to glutathione. After blocking the excess cross-linker with ethanolamine, the cross-linked beads were incubated with anti-serum at room temperature. The beads were subsequently washed with PBS, and the bound antibody was eluted by using 100 mM glycine pH 2.8. The pH of the eluate was adjusted to neutral immediately, and the eluted antibody was dialyzed, concentrated, quantified and stored at −80°C.

### *In vitro* transcription and translation

The *in vitro* transcription and translation of Myc–TNPO1 or Myc–Rab8-wt was conducted using TNT^®^ T7 Quick Coupled Transcription and Translation System (Promega) according to the manufacturer's protocol. The reaction mixture was incubated at 30°C for 90 min. The protein expression was verified by western blotting analysis.

### Immunoprecipitation and GST pull down

HEK293T cells were subjected to transfection as described above. After 24–36 h, cells were scraped into lysis buffer containing 50 mM HEPES pH 7.3, 150 mM NaCl and 1% Triton X-100, and the resulting lysate was cleared by centrifugation at 16,000 ***g*** at 4°C. The supernatant was incubated with ∼1 µg of antibody, 15 µl of GFP-Trap beads (ChromoTek) or 10–40 µg of GST fusion protein on glutathione beads for 4–14 h. When antibody was used, the antigen–antibody complex was subsequently captured using 15 µl of pre-washed Protein A/G beads (Pierce) for 2–4 h. Beads were washed with lysis buffer, and bound proteins were eluted by boiling in SDS sample buffer and resolved by performing 8–12% SDS-PAGE. The separated proteins were transferred to polyvinyl difluoride membrane (Bio-Rad). After incubation with primary and HRP-conjugated secondary antibodies, the chemiluminescence signal was detected by a cooled charge-coupled device camera (LAS-4000, GE Healthcare Life Sciences).

### Immunofluorescence microscopy

Cells were seeded on Φ12-mm coverslips (No. 1.5) in a 24-well plate. After 24 h of transfection, cells were serum starved to induce ciliogenesis and subsequently fixed with 4% paraformaldehyde in PBS at room temperature for 20 min. This was followed by neutralizing paraformaldehyde with 100 mM ammonium chloride and washing with PBS. The primary and secondary antibodies were diluted in fluorescence dilution buffer (PBS supplemented with 5% fetal bovine serum and 2% bovine serum albumin) containing 0.1% saponin (Sigma). Cells were incubated with primary antibody, washed and then incubated with a fluorescence-conjugated secondary antibody. After extensive washing, coverslips were mounted in Mowiol 4-88 (EMD Millipore). For surface labeling, cells grown on coverslips were incubated with CD8a monoclonal antibody on ice for 1 h. After washing with ice-cold PBS, cells were fixed and subjected to immunofluorescence labeling as described above. Cells were imaged under a wide-field microscope system comprising Olympus IX83 equipped with a Plan Apo oil objective lens (63× or 100×, NA 1.40), a motorized stage, motorized filter cubes, a scientific complementary metal oxide semiconductor camera (Neo; Andor Technology) and a 200-W metal-halide excitation light source (Lumen Pro 200; Prior Scientific). Dichroic mirrors and filters in filter turrets were optimized for GFP and Alexa-Fluor-488, mCherry and Alexa-Fluor-594 and Alexa-Fluor-647. The microscope system was controlled by using MetaMorph software (Molecular Devices), and only the center quadrant of the camera sensor was used for imaging.

### FRAP

RPE1 cells were seeded on Φ35 mm glass-bottomed Petri-dishes (MatTek) and transfected to co-express GFP-fused membrane reporters and Arl13b–mCherry (as a ciliary marker). After 24 h of transfection, cells were serum-starved to induce cilia. Live-cell imaging was conducted in CO_2_-independent medium (Invitrogen). Two-dimensional time-lapse images during FRAP were acquired by using a motorized Nikon Eclipse Ti microscope equipped with Plan-Apo oil lens (100×, NA 1.4), Perfect Focus System, Piezo *z*-stage, CSU-22 spinning disk scan head (Yokogawa), 37°C heated chamber (Lab-Tek), 100-mW diode lasers (491 nm and 561 nm), 3D FRAP system (iLAS^2^; Roper Scientific) and electron-multiplying charge-coupled device (Evolve 512; Photometrics). The microscope system was controlled by using MetaMorph and iLAS^2^ software (Roper Scientific). The two-dimensional time-lapse images of cilia were collected before and after photobleaching. Image analysis was conducted in ImageJ (http://imagej.nih.gov/ij/). The regions of interest (ROIs) of cilia were generated by using either intensity segmentation or manually drawn according to Arl13b–mCherry detection. The mean fluorescence intensity of ROIs at each post-photobleaching time point was fitted to a single exponential decay function y=y_0_+A_1_*exp(−(x−x_0_)/t_1_) using OriginPro8.5 (OriginLab). The immobile fraction was calculated as (I_pre_−y_0_)/(I_pre_−I_0_), where I_pre_ and I_0_ are the intensities before and immediately after photobleaching, respectively.

### Statistical analysis

All samples were randomly chosen and included in analyses once chosen. The sample size *n* is indicated wherever applicable in figures or corresponding legends. Data are presented as the mean±s.e.m. The two-tailed unpaired *t*-test analysis was conducted in Excel (Microsoft) and used for the analysis of statistical significance. A *P*-value of less than 0.05 was considered statistically significant. Linear regression fitting was performed in OriginPro8.5.
